# Structural and mechanistic insights into the bifunctional HISN2 enzyme catalyzing the second and third steps of histidine biosynthesis in plants

**DOI:** 10.1038/s41598-021-88920-2

**Published:** 2021-05-06

**Authors:** Wojciech Witek, Joanna Sliwiak, Milosz Ruszkowski

**Affiliations:** 1 Center for Biocrystallographic Research, Institute of Bioorganic Chemistry, Polish Academy of Sciences, Noskowskiego 12/14, 61-704 Poznan, Poland; 2 Synchrotron Radiation Research Section of MCL, National Cancer Institute, Argonne, IL USA

**Keywords:** X-ray crystallography, Hydrolases

## Abstract

The second and third steps of the histidine biosynthetic pathway (HBP) in plants are catalyzed by a bifunctional enzyme–HISN2. The enzyme consists of two distinct domains, active respectively as a phosphoribosyl-AMP cyclohydrolase (PRA-CH) and phosphoribosyl-ATP pyrophosphatase (PRA-PH). The domains are analogous to single-domain enzymes encoded by bacterial *hisI* and *hisE* genes, respectively. The calculated sequence similarity networks between HISN2 analogs from prokaryotes and eukaryotes suggest that the plant enzymes are closest relatives of those in the class of *Deltaproteobacteria.* In this work, we obtained crystal structures of HISN2 enzyme from *Medicago truncatula* (*Mt*HISN2) and described its architecture and interactions with AMP. The AMP molecule bound to the PRA-PH domain shows positioning of the N1-phosphoribosyl relevant to catalysis. AMP bound to the PRA-CH domain mimics a part of the substrate, giving insights into the reaction mechanism. The latter interaction also arises as a possible second-tier regulatory mechanism of the HBP flux, as indicated by inhibition assays and isothermal titration calorimetry.

## Introduction

Metabolic pathways have been the subject of extensive studies for more than a century. The study of L-histidine (hereafter histidine) biosynthesis in prokaryotes and lower eukaryotes has engaged scientists for nearly 70 years. The histidine biosynthetic pathway (HBP) was first studied on microorganisms, e.g., *Salmonella typhimurium* and *Escherichia coli*, and is well characterized in prokaryotes^1^. It unraveled many mechanisms fundamental to cell biology, e.g., an operon structure and gene expression^2^. Genetic and biochemical analysis of thousands of mutations in *his* operon in *S. typhimurium* showed that, in contrast to the fungus *Neurospora crassa*, the bacterial *his* genes were tightly clustered^3,4^. The observation of coordinated expression of that cluster led to the idea that a group of genes functions as a single unit of expression and regulation, today known as an operon^5,6^. Together with *lac*^7^ and *trp* operons^8^, *his* operon was used as a model system to study polar mutations^9^. Moreover, studies of the HBP helped to discover the regulation of amino acid biosynthesis by attenuation^10^.

The HBP is rather conservative among different domains of life; however, there are differences in the number of genes involved in the pathway and their expression pattern^11^. In bacteria, *his* genes are arranged in a compact operon (*hisGDC [NB] HAF [IE]*), with three of them (*hisD, hisNB* and *hisIE*) sometimes but not always coding for bifunctional enzymes^10,12^. Analysis of the structure of *his* genes revealed three main molecular mechanisms that are important in shaping the HBP, i.e., gene duplication, gene fusion, and gene elongation, which make this pathway a suitable model for understanding general molecular mechanisms behind metabolic routes^2^.

In plants, the HBP study was delayed (until the 1980s) due to a lack of genetic approach and complicated biochemistry standing behind the pathway. As a result, the first auxotrophic mutants in higher-plant systems arrived much later than their bacterial or fungal counterparts^13^. Recent progress in molecular biology techniques has revealed that many of the enzymatic steps of the HBP in plants are performed by proteins encoded by single genes, which is in contrast to the extensive gene redundancy found in other amino acid biosynthetic pathways in plants^14^. Genes encoding all eight histidine biosynthetic enzymes (*HISN1-8*) have been identified in *Arabidopsis thaliana*^15^. Five of the HBP enzymes in *A. thaliana* are encoded by single-copy genes, with duplications in *HISN1*, *HISN5,* and *HISN6*^16^.

**Figure 1:**
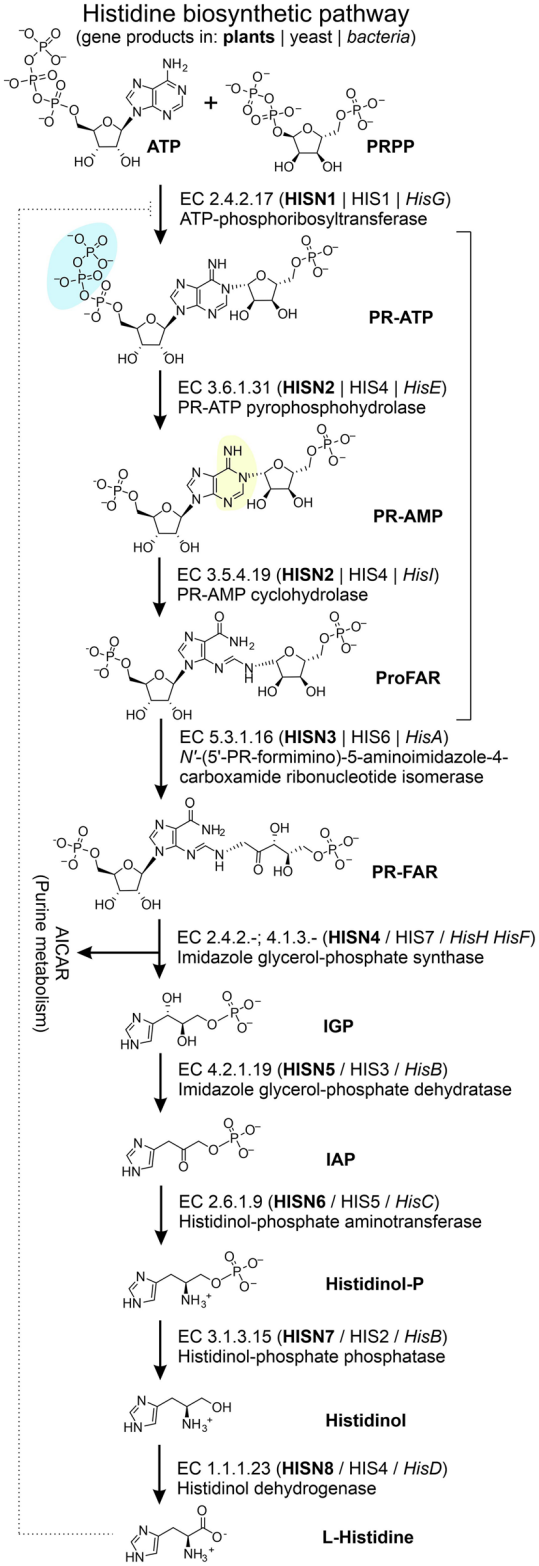
Organization of the histidine biosynthetic pathway (HBP) in plants, yeast, and bacteria. The large bracket marks the pathway fragment catalyzed by the PRA-PH and PRA-CH domains of *Mt*HISN2. Hydrolyzed groups by the PRA-PH and PRA-CH domains are highlighted by blue and yellow, respectively. The dotted line illustrates the feedback inhibition.

The HBP flux regulation at the post-translational level links to the first enzyme (HISN1), an ATP-phosphoribosyl transferase (ATP-PRT, EC 2.4.2.17, Fig. 1). ATP-PRTs catalyze condensation of ATP (adenosine-5′-triphosphate) and PRPP (phosphoribosylpyrophosphate) into PR-ATP (*N*^1^-5′-phosphoribosyl-ATP). ATP-PRTs are allosterically feedback-inhibited by histidine^17^. Furthermore, binding of adenosine-5′-monophosphate (AMP) at the active site increases the enzyme’s sensitivity to histidine, also in plants^18^. So far, there have been no implications that any other HBP enzyme could be regulated.

In the second step of the HBP, PR-ATP is hydrolyzed to *N*^1^-5′-phosphoribosyl-AMP (PR-AMP) by phosphoribosyl ATP pyrophosphohydrolase (PRA-PH; EC 3.6.1.31). In the third step, PR-AMP cyclohydrolase (PRA-CH, EC 3.5.4.19) opens the adenine ring of PR-AMP to produce *N*^1^‐[(5′‐phosphoribosyl)formimino]‐5‐aminoimidazole‐4‐carboxamide‐ribonucleotide (ProFAR). Then the HBP follows to yield histidine after eight more steps, catalyzed by subsequent enzymes.

Prokaryotes’ genomes often contain separate genes, *hisE* and *hisI,* that encode PRA-PH and PRA-CH enzymes, respectively^19^. However, in some bacteria, such as *E. coli* or *S. typhimurium*, the protein product of a fused gene, *hisIE*, has both activities. The gene fusion can go even further as, e.g., in yeast, *Saccharomyces cerevisiae,* a single gene (*HIS4*) encodes a trifunctional enzyme with activities of PRA-PH, PRA-CH, and histidinol dehydrogenase (HDH, EC 1.1.1.23)^20^. These are the second, third, and last reactions of the HBP, respectively (Fig. 1).

In the plant HBP, a single gene (*HISN2*) encodes a HISN2 enzyme that performs two subsequent reactions (Fig. 1). One domain of HISN2 has the PRA-PH activity, whereas the second domain has the PRA-CH activity^21,22^. In this study, we investigated the HISN2 enzyme from a model legume, *Medicago truncatula*, named *Mt*HISN2. The research focused on (i) the enzyme molecular structure, (ii) similarities and differences with bacterial orthologs of known structures, (iii) interactions with AMP, a proposed activity regulator, and (iv) the catalytic mechanism.

## Results and discussion

### Phylogenetic analysis suggests the evolutionary origin of plant HISN2 sequences

**Figure 2:**
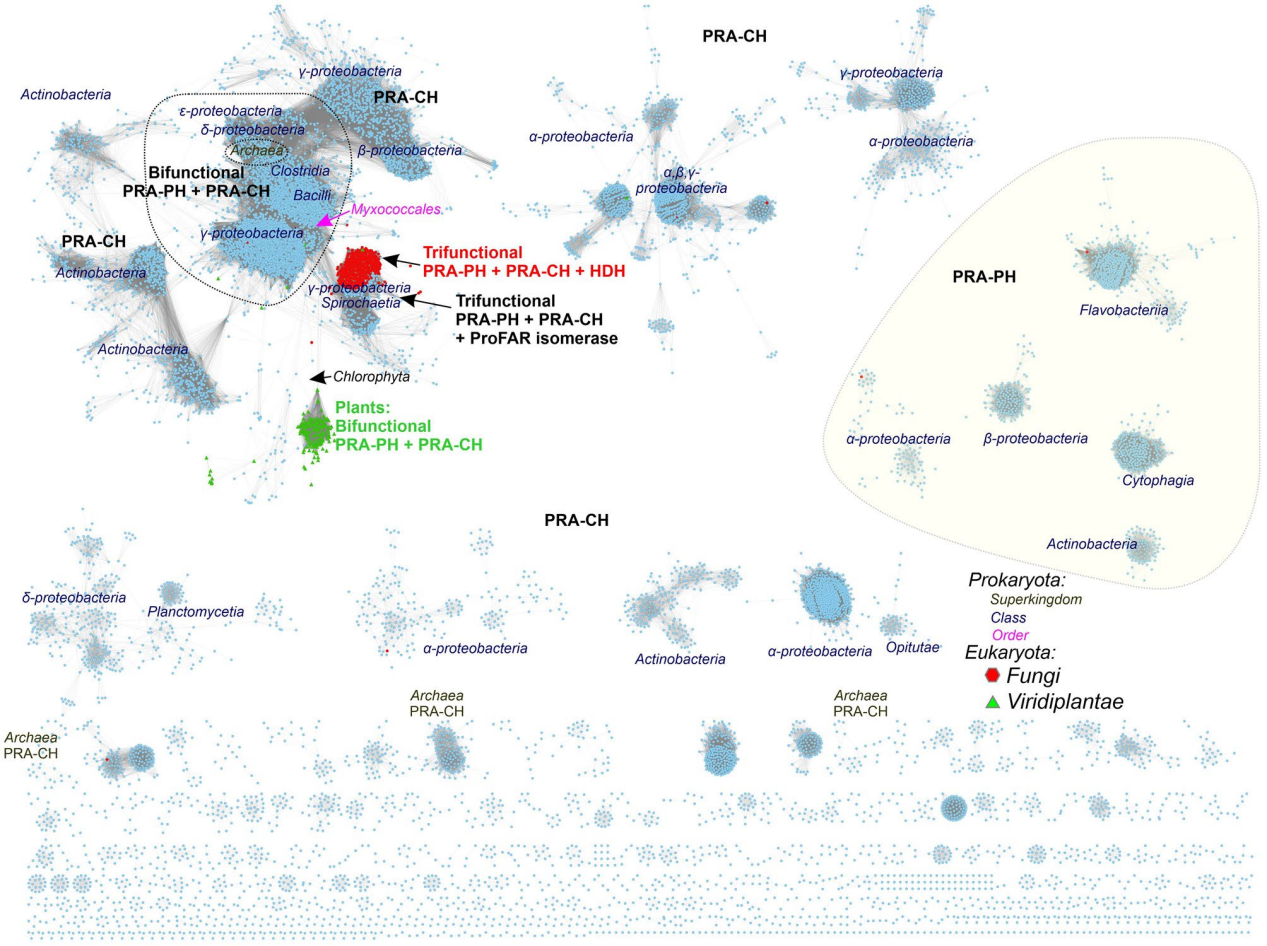
Sequence similarity network of PRA-PH and PRA-CH domains. UniRef90 sequences in InterPro families IPR008179, IPR021130, IPR002496, and IPR038019 were analyzed. 14 933 nodes are presented in the figure after rejecting 6748 outliers from the diagram. Sequences from *Eukaryota* are marked according to the legend. Monofunctional PRA-PH and PRA-CH proteins are most common in bacteria. Bi- and trifunctional enzymes are indicated with their specific activities. HDH, L-histidinol dehydrogenase; ProFAR, N^1^-(5′-phosphoribosyl-formimino)-5-aminoimidazole-4-carboxamide ribonucleotide.

We have analyzed 53 111 available sequences assigned to InterPro families IPR008179, IPR021130, IPR002496, and IPR038019 to assess the sequence similarity between prokaryotic and eukaryotic HISN2-equivalent enzymes and trace the evolution of plant HISN2 proteins. The analysis suggests that plant bifunctional enzymes derive from the *Myxococcales* order in the class of *Deltaproteobacteria* (Fig. 2). Fungal trifunctional proteins (HIS4 in yeast) with PRA-PH, PRA-CH, and HDH (histidinol dehydrogenase) activities also derive from orders close to *Myxococcales*. Moreover, sequences from some *Gammaproteobacteria* and *Spirochaetia* of PRA-PH, PRA-CH, and ProFAR isomerase activities seem to derive from a similar common ancestor. Multifunctional enzymes permit an optimal yield of gene expression without a need for additional transcription regulation, as noted in the genetic history of the HBP^16^. Aside from the multifunctional enzymes, most bacterial classes like *Alpha-, Beta-, Gamma-,* and *Deltaproteobacteria, Actinobacteria, Flavobacteria, Cytophagia,* and *Opitutae* express single-activity enzymes. Monofunctional enzymes are also common in the superkingdom of *Archaea*; however, there is a small group of archaeal species with bifunctional enzymes (Fig. 2).

As recently reported by Del Duca et al.^23^, gene elongation was a leading mechanism in the evolution of *hisA*, *hisF*, *hisB,* and *hisD* histidine biosynthetic genes. The hypothesis for their evolution was confirmed by high sequence similarities between two halves of the proteins and by structural and biochemical studies. Since sequences of the four enzymes encoded by those genes are highly conserved in prokaryotic and eukaryotic organisms, it is most likely that the gene elongation occurred in the early stage of HBP evolution, before the Last Universal Common Ancestor^23^. The diversity in *hisI/E* (bacteria), HIS4 (fungi), and HISN2 (plants) may be another example of the importance of the gene elongation and duplication that occurred at the very early stage of the HBP evolution.

### The overall structure of *Mt*HISN2: a dimeric enzyme with discrete and directly interacting pyrophosphohydrolase and cyclohydrolase domains

The complete sequence of *Mt*HISN2 contains 283 amino acid residues (UniProt ID^24^: A0A072U2X9; Gene: 25498966). All plant enzymes of the HBP are encoded by the genomic DNA and contain N-terminal chloroplast-targeting signal peptides^22^. In *Mt*HISN2, bioinformatic analysis with TargetP^25^ suggested the signal peptide encompasses approx. forty N-terminal residues. In *A. thaliana* HISN2, the target peptide spans fifty residues (UniProt ID: O82768). We designed the construct to include sequence conserved in plant species; hence our final construct starts from Val49, preceded by a linker tripeptide, Ser-Asn-Ala.

The X-ray structure of *Mt*HISN2 was solved by experimental phasing using single-wavelength anomalous dispersion (SAD) on zinc cations bound to the protein. The unliganded protein (with metals) crystallized in the *C*2 space group (Table 1) with two protein chains in the asymmetric unit (ASU). *Mt*HISN2-AMP complex crystallized in the *C*2 space group but with different unit cell parameters (Table 1) and six protein chains (three dimers) in the ASU. The obtained electron density maps allowed us to trace most of the protein chain unambiguously, except for up to eighteen C-terminal residues and fragments between 157–165 and 186–194 (model- and chain-dependent) that were disordered.

**Table 1:** Diffraction data and refinement statistics.

	*Mt*HISN2		*Mt*HISN2-AMP		
**Data collection**					
Wavelength (Å)	0.9793		1.0000		
Space group	*C*2		*C*2		
**Unit cell parameters**					
*a, b, c* (Å)	172.1, 69.3, 52.0		202.9, 68.6, 135.6		
β (°)	94.7		128.8		
Resolution cut-off method	Isotropic		Anisotropic		
Resolution shell	All	Outer	All	Inner	Outer
Resolution (Å)	80–1.60	1.70–1.60	48.45–2.70	48.45–7.83	2.85–2.70
Unique reflections	79,751	12,569	33,411	5848	6725
Multiplicity	3.7	3.8	3.9	3.5	4.1
Ellipsoidal completeness (%)	N/A	94.1	99.2	55.8	
Spherical completeness (%)	98.5	96.3	85.0	99.2	26.8
*R*_merge_ (%)	4.5	62.4	9.1	3.0	73.2
< *I*/σ(*I)* >	14.6	1.9	11.1	28.7	2.0
CC(1/2)	99.9	69.5	99.7	99.9	67.5
**Refinement**					
*R*_free_ reflections	1037		1010		
No. of atoms (non-H)	3701		10,004		
Protein	3336		9658		
Water	356		62		
Other	9		284		
*R*_work_/*R*_free_ (%)	14.3/17.5		18.1/24.6		
**RMSD from ideal geometry**					
Bond lengths (Å)	0.011		0.006		
Bond angles (°)	1.014		0.771		
**Ramachandran statistics (%)**					
Favored	97.8		98.9		
Allowed	2.2		1.1		
Outliers	0.0		0.0		
PDB ID	7BGM		7BGN		

**Figure 3:**
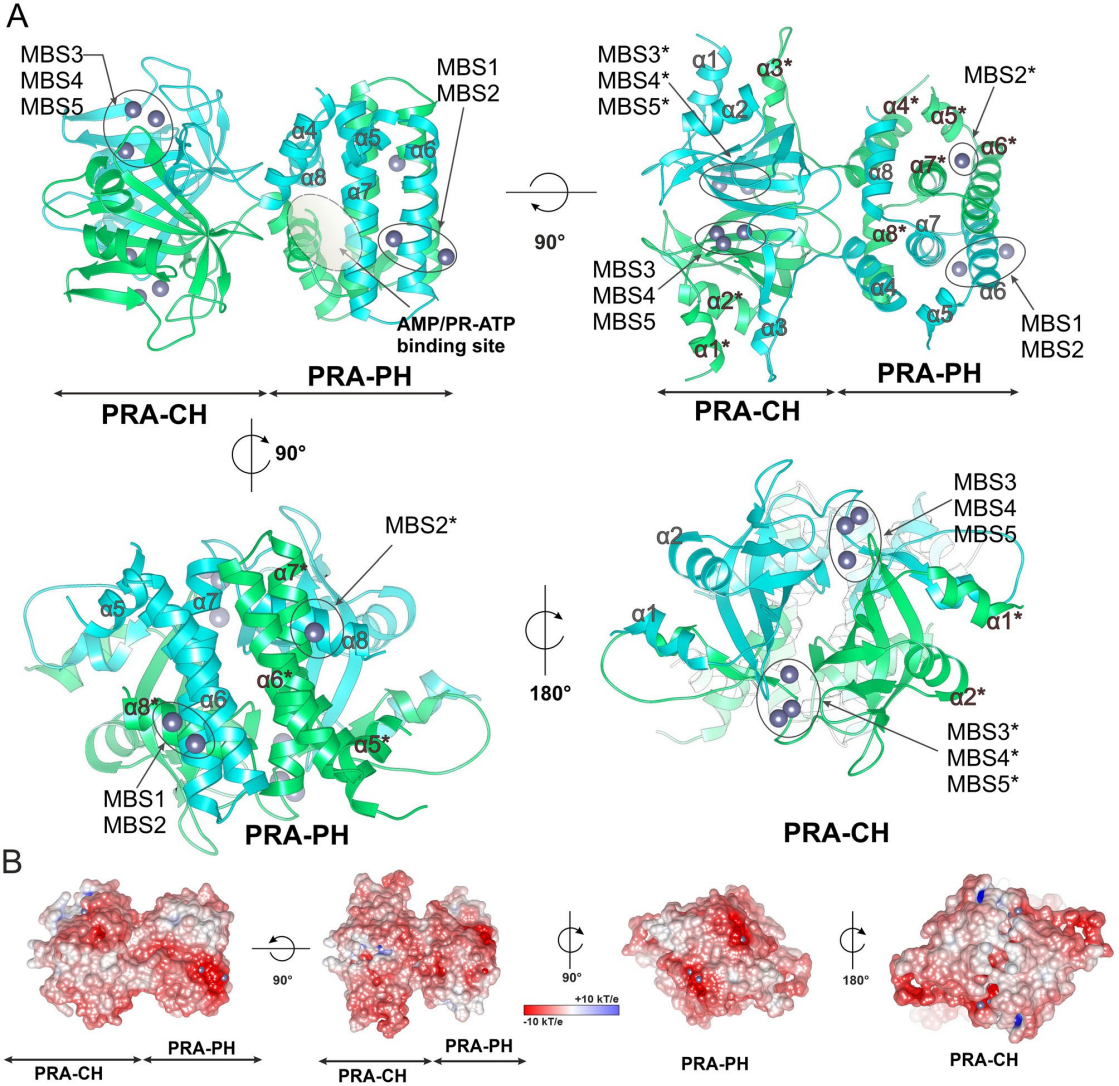
Structure of unliganded *Mt*HISN2. (**A**) Ribbon representation of the *Mt*HISN2 dimer; the metal-binding sites (MBSs) containing Zn^2+^ (dim gray) are marked in elipses; the AMP/PR-ATP binding site in the PRA-PH domain is marked in a dashed elipse. Notice overlapping chain A (light green) and chain B (cyan) that form well-separated domains of PRA-PH and PRA-CH activities. Asterisks (*) represent elements of symmetric subunits. (**B**) Surface electrostatic potential of *Mt*HISN2 is color-coded as shown in a bar. Protein rotations correspond to panel **A**, respectively.

*Mt*HISN2 forms a tight dimer of 26.4 kDa subunits (Fig. 3A), sharing a ~ 4000 Å^2^ interface, according to PISA analysis^26,27^. The dimeric form is consistent with the size-exclusion elution profile (not shown). The dimer’s surface area is ~ 20,000 Å^2^ and is negatively charged (Fig. 3B), agreeing with the calculated pI of 5.3. The negative charge suggests that metal cations play an important role in interactions with negatively charged, phosphate-containing substrates, PR-ATP and PR-AMP.

The enzyme dimer is formed by two mutually swapped polypeptide chains, forming a bilobial protein—each domain forms one lobe (Fig. 3). Per sequence analogy to corresponding enzymes from bacteria and other plant species, those domains catalyze PRA-PH and PRA-CH reactions (Fig. 1). The PRA-CH domain is located at the N-terminus, spanning residues 49–158 (Fig. 3). The PRA-PH domain lies at the C-terminus, ranging from residues 172–283. It must be noted here that in this article, we treat a domain as a complete and functional dimeric entity—with two active sites. The existence of a monomeric form of either PRA-PH or PRA-CH domain is highly improbable as it would expose vast hydrophobic regions. In *Arabidopsis*, both domains, apparently as dimers, were shown as functionally independent, even when expressed separately^28^.

**Figure 4:**
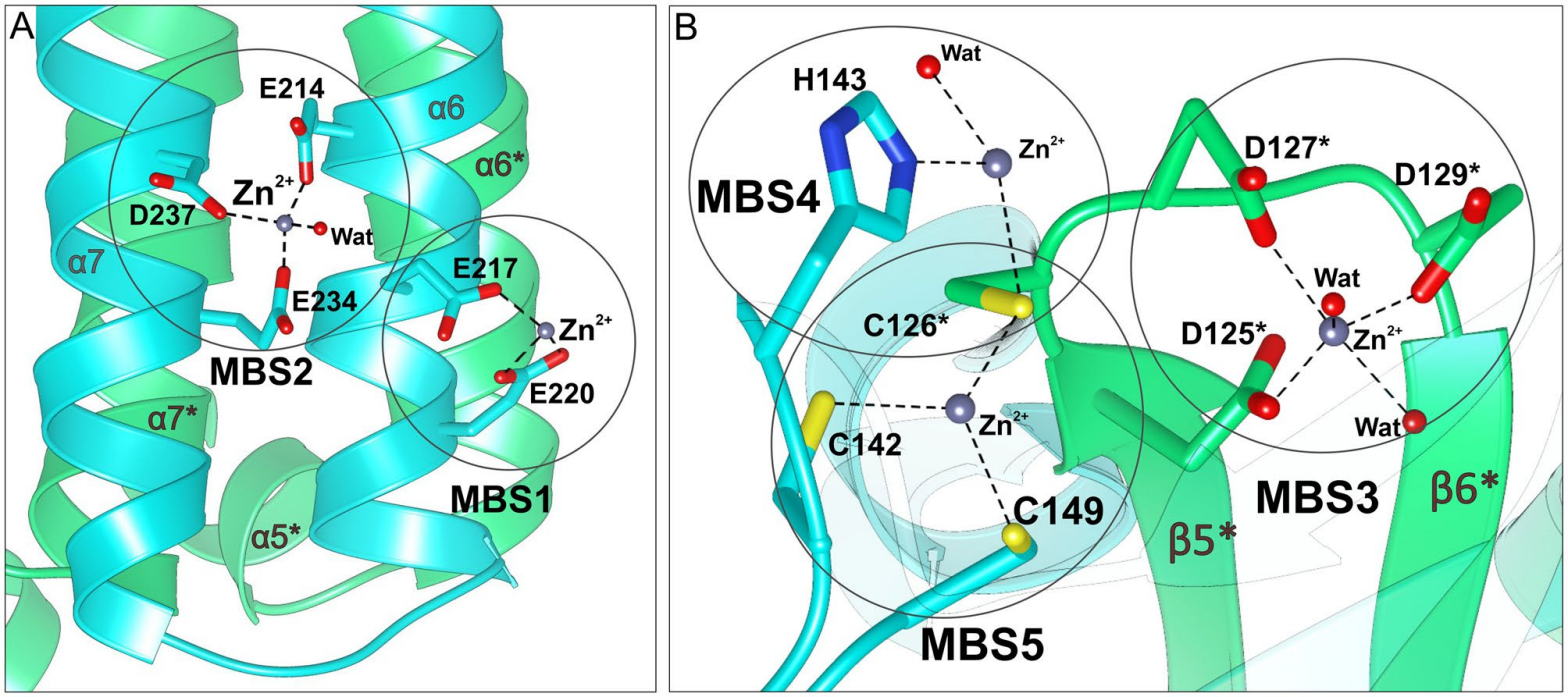
Metal-binding sites (MBSs) of *Mt*HISN2 in the unliganded form. Panel **A** shows zinc (dim grey) coordination by the residues of α6 and α7 and water molecules (red balls) in the PRA-PH domain. Orientation is the same as in the first panel in 3A. Panel **B** shows metal binding sites in the PRA-CH domain; in the AMP complex MBS4 is absent. Zinc in MBS3 is coordinated by residues of the loop connecting strands β5* and β6*. MBS4 and MBS5 are coordinated by residues of the chain B and Cys126* of the chain A.

The PRA-PH domain consists of two overlapping and swapped protein chains built entirely of α-helices connected by loops. Each chain of the domain contributes five α-helices (α4–α8, Fig. 3A). Helices α6 and α7 form a four-helix bundle with their counterparts from the dimer mate, α6* and α7* (an asterisk denotes an element from the other subunit within the dimer). Helices α6 and α7 contain the PRA-PH active sites, defined near the metal-binding sites 1 and 2 (MBSs, Fig. 4A). Except for the bundle consisting of the four longest helices, there are short helices α4 and α8 and their counterparts α4*, α8* that overlap on top of each other, creating a tight chain swap. The swap separates the four-helix bundle from the PRA-CH domain.

In general, PRA-PH enzymes are Mg^2+^-dependent^29^. However, the *Mt*HISN2 crystals could only be grown in the presence of a low concentration of Zn^2+^ in addition to Mg^2+^. Zinc often binds to proteins at non-specific sites or at sites naturally binding other metals^30^, which likely was the case here. Thus, we decided to use a more general term—MBSs—in this work to avoid confusion. There are two unique MBSs in the PRA-PH domain. MBS1 contains Zn^2+^ coordinated by two carboxyl oxygen atoms of Glu220 and one carboxyl oxygen of Glu217 (Fig. 4A). In MBS2, Zn^2+^ is tetrahedrally coordinated by carboxylic groups of Glu214, Glu234, Asp237, and a water molecule. In some subunits, Glu217 also participates in Zn^2+^ coordination in MBS2—resulting in the disappearance of MBS1. Because metal at MBS1 was absent in some subunits in our structures, only MBS2 may be catalytically relevant. Also, it is very likely that in vivo Mg^2+^ cations (not Zn^2+^) occupy MBS2, as magnesium, not zinc, is required for PRA-PH activity^31^.

The PRA-CH domain also consists of two overlapping chains but has an entirely different structure (Fig. 3A). The domain connects with the PRA-PH domain via two long loops consisting of twelve residues (159–171), each belonging to one chain. The core of the PRA-CH domain is made of β-strands and α-helices forming the so-called barrelizing β-grasp fold (β-GF), wherein the β-sheet “grasps” an α-helix in a fasciclin-like assemblage^32^. There are many kinds of β-GF, but all of them share a similar topology, where β-strands form a mixed β-sheet surrounding a helix (α2 in *Mt*HISN2). The most characteristic feature of the core four-stranded β-sheet is that the flanking strands are parallel to each other, while the two middle strands are anti-parallel to the flanking strands. This means that the first and the last strands (by sequence) are located in the central part of the sheet with a cross-over via an α-helical fragment. Variety of unrelated proteins where the β-GF was found indicates that, despite its relatively small size, the β-GF is a multifunctional scaffold suited for small-molecule binding (PR-AMP in this case). In *Mt*HISN2, a β-strand is followed by a helix and a loop that together form a super-secondary motif responsible for the domain swap. The β-sheet is connected with the motif via a long loop spanning residues 138–149 and contains residues coordinating MBSs 4–5 (see, Fig. 4B).

In our structures, the PRA-CH domains contain two or three (model- and subunit-dependent) MBSs that bind metal cations through conserved aspartate (Asp125*, Asp127*, and Asp129* in *Mt*HISN2), cysteine (Cys126*, Cys142, Cys149), and histidine (His143) residues (Fig. 4B). As noted by D’Ordine et al.^33^, corresponding residues are universally conserved in cyclohydrolases. In the PRA-CH structure from *Methanobacterium thermoautotrophicum,* the aspartate residues (Asp85, Asp87, and Asp89) coordinated Cd^2+^ in a site corresponding to the MBS3 of *Mt*HISN2 (Asp125, Asp127, and Asp129, respectively), where Zn^2+^ was bound^33^. The MBS3 is formed by the carboxylic groups of Asp125*, Asp127*, Asp129* and by two water molecules in a shape of a trigonal bipyramid. In the next site, MBS4, Zn^2+^ is coordinated tetrahedrally by two water molecules and Nε of His143 and by the thiol of Cys126*. However, we did not observe a metal cation bound at MBS4 in the *Mt*HISN2-AMP complex, suggesting that a metal bound to the MBS4 can either promote substrate binding or may not be physiologically relevant. Lastly, Zn^2+^ bound in the MBS5 is coordinated by thiols of Cys126*, Cys142, and Cys149 (Fig. 4B). Considering the chemical nature of residues in the metal coordination spheres (metal ligands), it is likely that Zn^2+^ occupies only MBS5 in vivo*,* while MBS3 and MBS4 may bind a cocatalytic Mg^2+^, per definition by Valle and Auld^34^. This hypothesis is consistent with the results of chemical probing of *Methanococcus vannielii* PRA-CH enzyme, which showed only one high-affinity Zn^2+^ binding site (corresponding to MBS5) per subunit^33^.

### Structural alignment of *Mt*HISN2 and its bifunctional bacterial counterpart reveals differences in the enzyme architecture while individual domains are similar

Structural comparisons of bacterial PRA-PH enzymes revealed high structural similarity, despite low sequence identities^29^. For instance, sequence identity as low as 31% between HisE from *Mycobacterium tuberculosis* (*Mtb*HisE, PDB ID: 1Y6X) and *Chromobacterium violaceum* HisE (CvHisE, 2A7W) still results in a very similar three-dimensional structure. BLAST sequence alignment between *Mtb*HisE and *Mt*HISN2 shows no significant similarity; however, both proteins share similar architecture in secondary structure topology, chain swaps, and the four-helix bundle.

The structural similarity despite relatively low sequence identity applies to the PRA-CH domain as well, as reflected by the RMSD of 0.68 Å between the *Mt*HISN2 PRA-CH domain and HisI of *M. thermoautotrophicum*, sharing sequence identity of 40%. As pointed by D’Ordine et al., alignment between archaeal, bacterial, and eukaryotic sequences, e.g., *M. thermoautotrophicum*, *E. coli*, *S. cerevisiae,* reveals that some residues are highly conserved among PR-AMP cyclohydrolases^33^, which is consistent with their role in metal coordination also in *Mt*HISN2.

**Figure 5:**
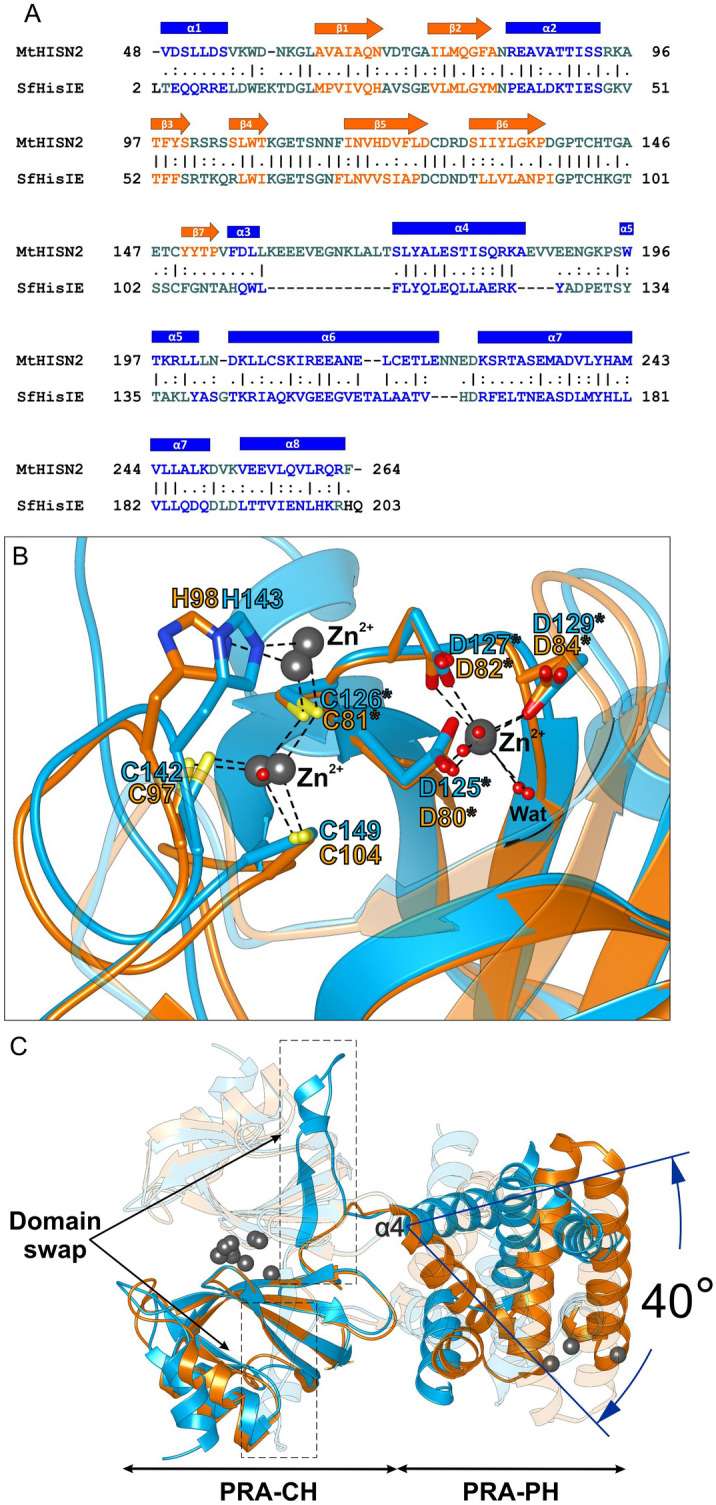
Comparison of a bacterial bifunctional HisIE enzyme and plant *Mt*HISN2. Panel **A** shows a sequence alignment of *Mt*HISN2 and *Sf*HisIE (35% identity; PDB ID: 6J2L). Secondary structure elements are colored in blue (α-helices) and orange (β-strands). **B** Superposition of MBSs in the PRA-CH domains of *Mt*HISN2 (blue) and the *Sf*HisIE (orange), made up by evolutionary conserved residues coordinating Zn^2+^ (dim gray). Superposition of the two structures in panel **C** reveals well-aligning PRA-CH domains, whereas PRA-PH domains are rotated by 40°, measured between the α4 helix in *Mt*HISN2 and its counterpart in *Sf*HisIE. The second subunits of both dimers are transparent for clarity.

So far, the only structure of a bifunctional HisIE enzyme has been determined for *Shigella flexneri Sf*HisIE (PDB ID: 6J2L)^35^. Sequences of *Mt*HISN2 and *Sf*HisIE share 35% identity and 51% similarity, which indicates relatively low conservation. However, *Sf*HisIE has a similar topology to *Mt*HISN2 and lacks only the β7 strand and the α5 helix (in *Mt*HISN2 topology). The *Sf*HisIE sequence has three gaps, corresponding to residues 159–171, 185–188, 223–225 in *Mt*HISN2 (Fig. 5A).

Despite *Mt*HISN2 and *Sf*HisIE are somewhat distant homologs, their structural alignment reveals significant similarity in both individual PRA-PH (RMSD of 0.90 Å) and PRA-CH domains (RMSD of 0.84 Å). For instance, the PRA-CH active site of *Sf*HisIE and *Mt*HISN2 share a very similar architecture (Fig. 5B). However, significant differences arise from the comparison of the entire enzyme molecules. When the PRA-CH domains are superposed, relative rotations of the PRA-PH domains, measured as the axis of the α4 helix, differ by ~ 40° (Fig. 5C). Another major difference is the presence of a super-secondary strand-helix-loop motif near the domain-domain interface in the plant enzyme. It encompasses residues 150–172 of the *Mt*HISN2 sequence, which correspond to 105–110 in *Sf*HisIE. In *Mt*HISN2, it is involved in domain swapping by mutually overlapping corresponding chains, whereas *Sf*HisIE lacks that motif entirely (Fig. 5C). In summary, most differences between *Mt*HISN2 and *Sf*HisIE appear near or at the inter-domain junction.

### The architecture of *Mt*HISN2 indicates that PR-AMP intermediate is released between the two catalytic events

The protein structure was investigated using CAVER 3.0^36^ PyMOL Plugin in the context of possible tunnels that may connect active sites of PRA-PH and PRA-CH domains to shuttle the PR-AMP intermediate. Such tunnels are common in hydrolases, including two-domain hydrolases^37–39^. In *Mt*HISN2, none of those tunnels would allow the transport of molecules—even as small as water—between the catalytic sites. We note that in some cases, such tunnels appear after binding of small molecules that change the overall shape of a protein; however, (i) we did not detect any conformational changes in the enzyme, and (ii) the diameter of the narrow fragment between the domains is only ~ 15 Å wide. This excludes the possibility of moving the PR-AMP intermediate between the catalytic sites. Because the catalytic sites of both domains are > 40 Å apart, PR-AMP must diffuse to the solvent (chloroplast stroma) after pyrophosphate cleavage to reach the PRA-CH domain. This also means that after being produced by the PRA-PH domain, PR-AMP molecules may be processed further by a PRA-CH domain in a different enzyme molecule.

### AMP binding to the PRA-PH domain: positioning of the PR-ATP N1-phosphoribosyl

Our *Mt*HISN2-AMP complex showed that the enzyme active sites are adapted to bind nucleotides despite the lack of super-secondary structures typical for such specificity. More precisely, there are no Rossmann-fold motifs, often associated with cofactors like FAD, NAD^+^, and NADP^+^, or Walker motifs, commonly present in ATP-binding proteins^40,41^. The previous analysis of *Sf*HisIE also did not reveal Rossmann fold and Walker motifs^35^. In the *Mt*HISN2-AMP complex, AMP molecules were found near MBSs in both domains, PRA-PH and PRA-CH. For clarity, representative AMP molecules with the lowest B-factors are described.

**Figure 6:**
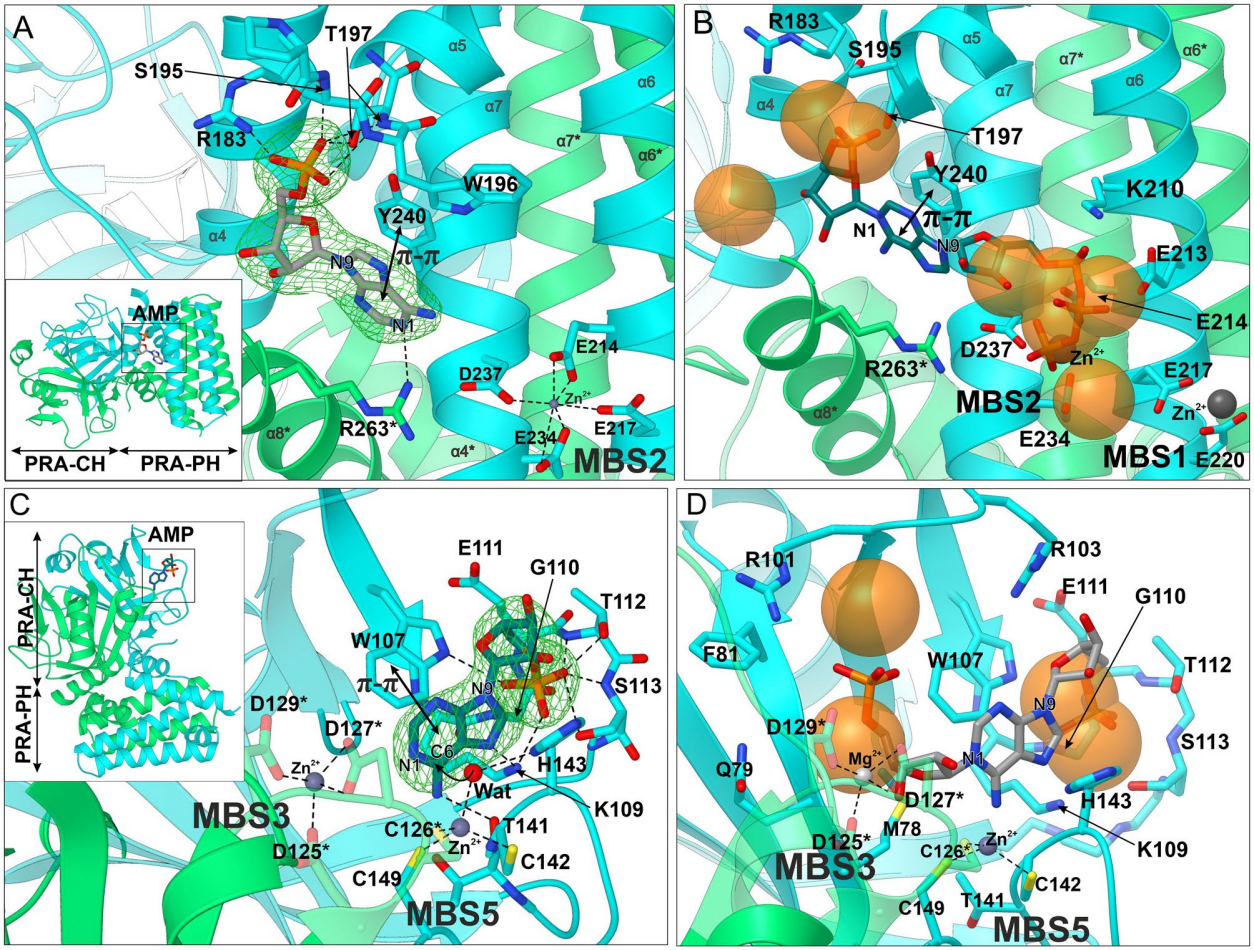
Interaction of AMP with *Mt*HISN2 and in silico docking of PR-ATP and PR-AMP. AMP binding to the PRA-PH domain (chain A) is shown in panel **A**. AMP was bound near MBS2 and coordinated by electrostatic interactions and π–π stacking between the side chain of Y240 and adenine ring of AMP. *F*_o_–*F*_c_ polder maps (green mesh) are contoured at 5σ level. Panel **B** illustrates in silico prediction of PR-ATP (dark cyan) binding, performed in AutoDock Vina. Sidechains of residues within a 5-Å radius are shown. Transparent orange balls from the analysis by the Nucleos server indicate areas of the highest probability of phosphate binding. Note that the triphosphate of docked PR-ATP aligns well with the prediction of multiple phosphate positions near MBS2. (Panel **C**) shows AMP binding to the PRA-CH domain. AMP was bound near MBS5 by electrostatic interactions, H-bonds, and π–π T-shaped stacking. The catalytic water molecule, activated by Zn^2+^ at MBS5 and His143, performs the nucleophilic attack (curvy arrow). The PR-AMP docking pose (obtained and presented as in panel **B**), compatible with the AMP binding mode, is shown in panel **D**. The metal cation at MBS3 was changed to Mg^2+^ as it appears more relevant in vivo.

AMP bound in the PRA-PH domain formed hydrogen bonds through the phosphate moiety and the adenine ring (Fig. 6A). The guanidine group of Arg183 formed polar hydrogen bonds with one oxygen of the phosphate. The second oxygen of the phosphate group interacted with the hydroxyl groups of Ser195 and Thr197 and with the backbone amide of Thr197. The backbone amide of Trp196 contacted the third phosphate oxygen. The adenine N1 atom interacted with the Arg263 guanidine group. We also observed the π-π stacking between the adenine ring and the Tyr240 side chain; the approximate inter-ring distance was 3.6 Å (Fig. 6A).

In that context, we note that AMP bound to the PRA-PH domain in our structure most likely does not show a part PR-ATP (substrate) or PR-AMP (product). This conclusion is based on the orientation of the AMP phosphate group pointing away from the metal center (MBS1-2) and interacting with the guanidine group of Arg183 instead. In contrast, the ATP fragment of PR-ATP should have its triphosphate group near the metal center for the hydrolysis to occur. To gain more insights, we utilized two in silico methods in parallel. We analyzed putative phosphate-binding regions in the *Mt*HISN2 structure using Nucleos^42^. It indicated that more phosphate groups (e.g., triphosphate) could bind near the MBS1-2 sites rather than near Arg183 (Fig. 6B). Molecular docking of PR-ATP with AutoDock Vina was consistent with the Nucleos results (Fig. 6B). The proposed orientation of the adenine ring of PR-ATP was rotated by ~ 180° in the ring’s plane to the AMP pose in the *Mt*HISN2-AMP complex. This means that the binding of AMP to the PRA-PH domain in our *Mt*HISN2 complex apparently shows the positioning of the N1-phosphoribosyl of PR-ATP and the plane of its adenine ring.

### AMP binding to the PRA-CH domain: an update to the catalytic mechanism

The second AMP binding site was located within the PRA-CH domain (Fig. 6C). The phosphate moiety formed an extensive network of hydrogen bonds with surrounding residues. The phosphate O1 atom bound to Nε of Trp107 and the backbone N of Gly110. The O2 atom interacted with the hydroxyl group and the backbone N of Ser113 and the hydroxyl group and backbone N of Thr112. The O3 atom was bound to the hydroxyl group of Ser113, the amine group of Lys109, and a water molecule. Moreover, the adenine N6 atom interacted with the carbonyl of Thr141, whereas N7 H-bonded with the amine group of Lys109. We also observed edge-to-face interaction between the aromatic rings of the adenine and Trp107, with ≈ 3.5 Å distance and angle ω ≈ 45°.

As reported by D’Ordine et al.^33^, the in silico docking of PR-AMP to the PRA-CH enzyme from *M. thermoautotrophicum* indicated that the substrate molecule in the active site is bound mainly by eighteen residues of which sixteen are conserved, and one is preserved in all PR-AMP cyclohydrolases^33,43^. The authors proposed two phosphate-binding regions, (i) Ser60, Thr61 and Ser62 (Ser100, Arg101, Ser102 in *Mt*HISN2) for the N9-phosphoribosyl, and (ii) Glu71, Ser72 and Ser73 for the N1-phosphoribosyl (Glu111, Thr112, Ser113 in *Mt*HISN2). Another interaction predicted by the authors to assist in substrate recognition is edge-to-face interaction between the adenine ring and Trp67 (Trp107 in *Mt*HISN2). The N9 ribosyl group was proposed to interact with Mg^2+^ and Arg15, which has no corresponding residue in *Mt*HISN2. His110 (His143 in *Mt*HISN2) was predicted to have a role in catalysis and π-stacking with the incoming substrate molecule. In terms of N1- and N9-phosphoribosyl orientations, a similar model has been reported by Wang et al.^35^, who also used in silico PR-AMP docking.

The AMP position in our *Mt*HISN2-AMP complex does not agree with the previously-presented in silico models. Nevertheless, the *Mt*HISN2-AMP complex is the first experimental structure showing (at least) a part of the PR-AMP substrate in the PRA-CH active site. In the *Mt*HISN2-AMP complex, N9-phosphoribosyl interacts with the region formed by residues _107_WTKGETS_113_, suggesting that the PR-AMP pose would be rotated by ~ 180° in the adenine ring plane, compared to the model by D’Ordine et al.^33^. In consequence, the region formed by residues _100_SRS_102_, likely interacts with the N1-phosphoribosyl. It is also possible that MBS3 plays a role in binding the N1-phosphoribosyl, especially since Mg^2+^ bound to the corresponding site was essential for the activity of other PRA-CH enzymes^33,44^. Our AMP pose with the N6 atom pointing towards the protein core (and not the solvent) agrees with the complexes of adenosine deaminases, a family of Zn^2+^-dependent hydrolases acting on adenosine-like substrates^45,46^. We must also note that we observed C2′-*endo* ribose in the *Mt*HISN2-AMP complex, meaning that even AMP, lacking the N1-phosphoribosyl, already binds “contracted” to the PRA-CH active site. D’Ordine et al. acknowledged that dealing with the flexibility of ribose rings was a big challenge during docking^33^. In our docking experiments, PR-AMP was bound to the PRA-CH domain (Fig. 6D) in a pose that is compatible with that of AMP in the *Mt*HISN2-AMP (Fig. 6B, D).

**Figure 7:**
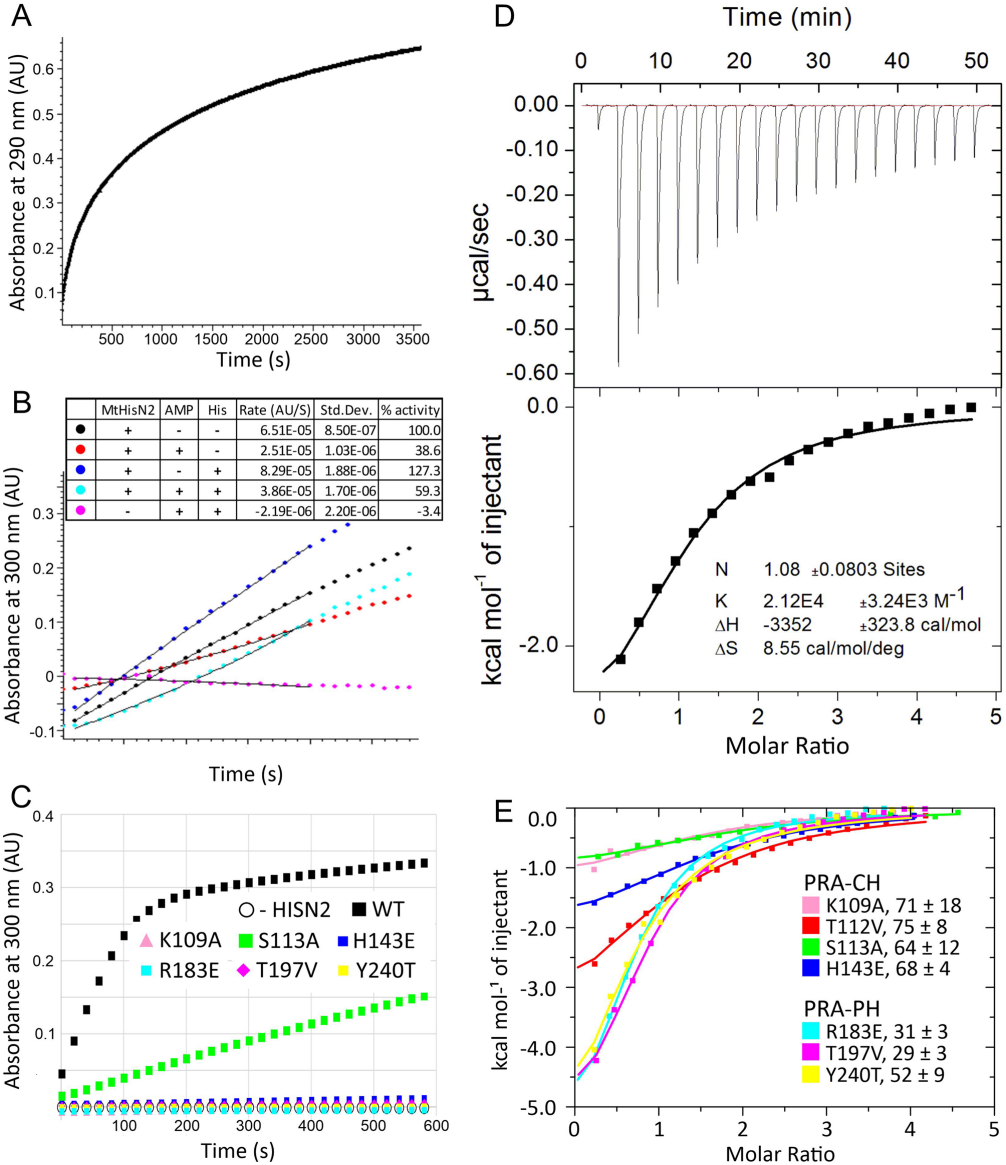
*Mt*HISN2 activity measurements. Panel **A** shows the course of enzymatic PR-ATP production. Results of the AMP (at 100 μM concentration) inhibition assay, in the presence and absence of histidine (100 μM), are presented in panel **B**; ProFAR increase was monitored at 300 nm. Panel **C** illustrates relative activities of the wild-type *Mt*HISN2 and its point mutants. Microcalorimetric study of the interaction between *Mt*HISN2 and AMP is shown in panels **D–E**. Representative ITC results for the wild-type protein is shown in panel **D**; the raw data are in the upper part, while the bottom part shows the best fit of one sets of binding sites model to the integrated peaks. Panel **E** shows AMP binding properties of the *Mt*HISN2 point mutants; *K*_d_ values ± errors are shown for each mutant in μM.

Thanks to the conserved three-cysteine active site (Cys142, Cys149, and Cys126*, MBS5), the general PRA-CH mechanism may be adopted from other reports^33,47^ and updated by the experimental position of AMP, which mimics a part of PR-AMP (Fig. 6C,D). First, PR-AMP is oriented in the catalytic pocket by the two phosphate-binding regions, namely (i) N1-phosphoribosyl orients towards _100_SRS_102_ and/or Mg^2+^ coordinated by Asp125*, Asp127*, and Asp129*, while (ii) N9-phosphoribosyl attracts to _107_WTKGETS_113_. The adenine moiety is secured by a hydrogen bond between its N7 atom and Nζ amine of Lys109 and by the edge-to-face interaction with Trp107. The nucleophilic water molecule in the Zn^2+^ coordination sphere (MBS5) is activated by His143, acting as a general base. A metal cation (MBS4) may play a role in priming His143; in the unliganded *Mt*HISN2 structure, His143 does not bind a water molecule but instead is in the MBS4 coordination sphere (Fig. 4B). The activated water molecule (or rather a hydroxyl anion) performs a nucleophilic attack on the purine C6 atom, breaking the N1-C6 bond. Distances observed in the *Mt*HISN2-AMP complex, Zn^2+^…H_2_O of 2.4 Å, Nδ of His143…H_2_O of 3.0, and H_2_O…C6 of 3.1 Å, are consistent with this mechanism. The role of the His143 as the general base is supported by lack of detectable activity of the H143E mutant, while a (weaker) binding of PR-AMP may still occur, as deduced from the *K*_d_ for AMP of 68 μM (Fig. 7). Moreover, environment of the active site pocket suggests that the optimal positioning of N1-phosphoribosyl may stretch the substrate, aiding the ring hydrolysis (Fig. 6D).

### AMP is an inhibitor of the PRA-CH domain of *Mt*HISN2 at physiologically-relevant concentrations

AMP is an activity regulator of plant HISN1 enzymes and their counterparts from other kingdoms of life. Although it has been shown that AMP alone does not exhibit an inhibitory effect on *Mt*HISN1, it significantly increases sensitivity to feedback regulation by free histidine^18^. However, so far, there have been no indications that other HBP enzymes could be regulated by AMP. In this work, *Mt*HISN2 inhibition by AMP was assayed using PR-ATP produced enzymatically, as PR-ATP is commercially unavailable. The PR-ATP production, prior to *Mt*HISN2 measurements, was monitored spectrophotometrically (at 290 nm, Fig. 7A). That mixture was then used to trigger AMP inhibition assays with *Mt*HISN2, in which the PR-ATP concentration was 18 μM, so that absorbance changes (at 300 nm) could be monitored^44^. Since ATP-PRT enzyme was still present in the *Mt*HISN2 reaction mixture, we also cross-validated the assay by including free histidine (at 100 μM), known to inhibit ATP-PRTs. We observed that 100 μM AMP caused over 60% inhibition. It must also be noted here that the AMP concentration, for instance, in maize chloroplasts ranges from 40 μM to 260 μM^48^. This puts *Mt*HISN2-AMP interaction as a possible secondary regulation mechanism of the HBP flux. Unfortunately, to our knowledge there is no data on the PR-ATP concentration in vivo. Notwithstanding, the 18 μM concentration used in our assay may even be exaggerated, as PR-ATP is readily processed by the HBP. Interestingly, when both AMP and histidine were present, the *Mt*HISN2 inhibition was mitigated to 41% (Fig. 7B). Because ATP-PRT enzymes bind AMP in the presence of histidine, the pool of AMP available to bind to *Mt*HISN2 decreases, providing the most likely explanation to this phenomenon. The control sample, without *Mt*HISN2, excluded the impact of the ATP-PRT reaction on the observed absorbance change at 300 nm at the moment of the HISN2 reactions, which were run simultaneously.

AMP interaction with *Mt*HISN2 in solution was further investigated using isothermal titration calorimetry (ITC). Our data show that AMP binding to *Mt*HISN2 (Fig. 7D) is characterized by the *K*_d_ value of 47 ± 6 µM and stoichiometry N = 1. Thermodynamic parameters are ΔH = -3352 ± 324 cal/mol and ΔS = 8.6 cal/mol/deg. To deduce whether the obtained *K*_d_ can be attributed to AMP binding to the PRA-PH or to the PRA-CH domain, we performed ITC experiments on point mutants of *Mt*HISN2. Four mutants within the PRA-CH domain (K109A, T112V, S113A, and H143E) and three within the PRA-PH domain (R183E, T197V, and Y240T) were tested and the results are shown in Fig. 7E. The results clearly indicate that the AMP binding affinity is lowered in the case of PRA-CH domain mutants. Moreover, these mutations significantly lower the heat effect of AMP binding in comparison with PRA-PH domain mutants (Fig. 7E). These two observations indicate that AMP binding to the PRA-CH domain is driven by enthalpy, thus can be measured by ITC. We cannot also exclude an auxiliary impact of AMP binding to the PRA-PH domain on the overall *Mt*HISN2 activity.

## Conclusions and outlook

This article is the fifth in a series of papers that show the structures of plant HBP enzymes. Previous structures were reported for: HISN1^18^, HISN5^49^, HISN7^50^, and HISN8^51^. In this work, we experimentally solved the structure of the HISN2 enzyme from the model legume, *Medicago truncatula* using X-ray diffraction data. The bifunctional *Mt*HISN2, with distinct PRA-PH and PRA-CH domains, showed significantly different relative orientation of the domains than in bacterial enzymes. Comparing bacterial and plant enzymes shed new light on the possible design of small-molecule inhibitors as potential antibiotics or herbicides. In this perspective, HisI, HisE, (or HisIE), homologs of fungal HIS4, and plant HISN2 enzymes may arise as promising molecular targets. If one wants to target bacterial or plant enzymes specifically, regions other than the conserved active sites appear most auspicious. The proposed insights into the regulation and catalytic mechanism provide groundwork for the design of HISN2 inhibitors, in addition to bringing a deeper comprehension of the plant HBP.

*Mt*HISN2 interacts with AMP, as shown by our complex crystal structure, inhibition assays, and ITC experiments, which indicated that *Mt*HISN2 activity regulation occurs in a physiologically-relevant range of AMP concentration. This way, the HBP flux can be tightly controlled on two steps, catalyzed by HISN1 and HISN2 enzymes. The need to control the HBP flux rises from a high metabolic cost of the pathway, estimated as equivalent to over thirty ATP molecules^52^. The HBP is at the same time the only pathway of amino acid biosynthesis that utilizes carbon and nitrogen directly from ATP. As fluctuations of the AMP/ATP ratio reflect the cell metabolic status, an AMP-based control can regulate resource consumption by the HBP.

## Materials and methods

### Cloning, expression, and purification

The total RNA was isolated from young *M. truncatula* leaves using the RNeasy Plant Mini Kit (Qiagen). The following reverse transcription with oligo dT_18_ primer yielded the complementary DNA (cDNA). The chloroplast-targeting peptide was recognized using the TargetP 1.1 server^25,53^, and the produced construct was N-truncated at Val49. The desired fragment was amplified by polymerase chain reaction; primers used in this work are given in Table 2. The expression plasmid, based on the pMCSG68 backbone (Midwest Center for Structural Genomics), was created by the ligase-independent cloning method^54^. Mutagenic substitutions were conducted using the Polymerase Incomplete Prime Extenstion (PIPE) method^55^ on the wild-type *Mt*HISN2 expression plasmid as a template and primers listed in Table 2. Correctness of all inserts was confirmed by DNA sequencing.

**Table 2:** Primer sequences used in this work.

Primer name	Sequence
MtHISN2-WT-F	TACTTCCAATCCAATGCCGTAGACTCATTGTTGGACAGTGTAAAATG
MtHISN2-WT-R	TTATCCACTTCCAATGTTATCAATTTTCCACCGATTTCTGGGTTGG
K109A-F	GTTGTGGACCGCGGGAGAGACCTCCAATAATTTCATCAATGTC
K109A-R	GTCTCTCCCGCGGTCCACAACGATGATCGTGACC
T112V-F	GGAGAGGTGTCCAATAATTTCATCAATGTCCATGATGTC
T112V-R	GAAATTATTGGACACCTCTCCTTTGGTCCACAACGATG
S113A-F	GGAGAGACCGCGAATAATTTCATCAATGTCCATGATGTC
S113A-R	GAAATTATTCGCGGTCTCTCCTTTGGTCCACAACG
H143E-F	CCTACCTGCGAGACAGGGGCAGAAACATGCTACTATAC
H143E-R	GCCCCTGTCTCGCAGGTAGGCCCATCAGGTTTC
R183E-F	CAATATCCCAGGAGAAGGCAGAGGTAGTAGAAGAAAATGGAAAG
R183E-R	CTCTGCCTTCTCCTGGGATATTGTTGACTCTAATGCATACAG
T197V-F	CTTCATGGGTCAAGCGGTTATTGCTTAATGATAAGTTGC
T197V-R	CAATAACCGCTTGACCCATGAAGGCTTTCCATTTTCTTCTAC
Y240T-F	GATGTACTCACGCATGCCATGGTTCTGTTGGCACTG
Y240T-R	CATGGCATGCGTGAGTACATCAGCCATCTCTGAAGCAG

Overexpression was carried in BL21 Gold *E. coli* cells (Agilent Technologies) in LB media with 150 μg/mL ampicillin. After incubation with shaking at 190 rpm at 37 °C until the A_600_ reached 1.0, the cultures were chilled to 18 °C, and isopropyl-d-thiogalactopyranoside was added at a final concentration of 0.5 mM to start overexpression, which went on for 18 h. The cell pellet from the 2-L culture was centrifuged at 3500×*g* for 20 min at 4 °C and resuspended in 35 mL of binding buffer [50 mM Hepes–NaOH pH 7.5; 500 mM NaCl; 20 mM imidazole; 2 mM tris(2-carboxyethyl)phosphine (TCEP)] and stored at − 80 °C for purification.

The cells were disrupted by sonication (4 min with intervals for cooling), and the cell debris was removed by centrifugation at 25,000×*g* for 30 min at 4 °C. The supernatant was mixed with 3 mL of HisTrap HP resin (GE Healthcare) in a column on the VacMan setup (Promega). The resin-bound protein was washed five times with the binding buffer and eluted with 20 mL of elution buffer (50 mM Hepes–NaOH pH 7.5; 500 mM NaCl; 400 mM imidazole; 2 mM TCEP). The His_6_-tag was cleaved with TEV protease (at final concentration 0.2 mg/mL) overnight, simultaneously with dialysis to lower the imidazole concentration to 20 mM. The second run through the HisTrap resin resulted in pure *Mt*HISN2 in the flow-through to which ZnCl_2_ was added at 100 µM final concentration. The sample was concentrated to 2.4 mL and loaded on a HiLoad Superdex 200 16/60 column (GE Healthcare), equilibrated with buffer: 25 mM Hepes–NaOH pH 7.5, 100 mM KCl, 50 mM NaCl, 100 µM ZnCl_2_, and 1 mM TCEP. The protein was then concentrated and used for crystallization or functional assays.

### Crystallization, X-ray data collection, and processing

*Mt*HISN2 was crystallized using the vapor diffusion method. The protein concentration was 10 mg/ml, as determined by A_280_ measurement (molar extinction coefficient, ε of 43,430 M^−1^⋅cm^−1^). The unliganded structure results from crystals (hanging-drop) obtained by mixing 4 μl of the protein solution and 2 μl of 60% Morpheus D1 condition (Molecular Dimensions)^56^. The components of Morpheus D1 are: 0.12 M Alcohols (0.2 M 1,6-Hexanediol; 0.2 M 1-Butanol 0.2 M 1,2-Propanediol; 0.2 M 2-Propanol; 0.2 M 1,4-Butanediol; 0.2 M 1,3-Propanediol) 0.1 M Buffer System 1, pH 6.5 (Imidazole; MES-acid) 30% Precipitant Mix 1 (20% v/v PEG 500* MME; 10% w/v PEG 20,000). The crystals were cryoprotected by adding Morpheus D1 condition supplemented with 20% ethylene glycol. For the *Mt*HISN2-AMP complex structure, 10 mM MgCl_2_, 0.1 mM ZnCl_2_, and 20 mM AMP (added in 100 mM Hepes pH 7.5) were included in the solution subjected to crystallization. Then, the PEG/Ion screen (Hampton Research) supplemented with 7.5% glycerol was set up on a sitting-drop crystallization plate (1:1 μl mixtures). The crystals appeared in A11 condition (0.2 M potassium iodide, 20% Polyethylene glycol 3350). Immediately before crystal harvesting, 1 μl of PEG/Ion A11 condition with 50% of glycerol was added to the drop. All crystals were vitrified in liquid nitrogen and stored for synchrotron data collection.

Diffraction data were collected at the SER-CAT beamline 22-ID and SBC 19-ID at the Advanced Photon Source, Argonne National Laboratory, USA. Diffraction data were processed with the XDS package^57^. Anisotropic truncation of X-ray data for the *Mt*HISN2-AMP complex was done using the STARANISO server^58^. Data processing statistics are given in Table 1.

### Determination and refinement of the crystal structures

The crystal structure of *Mt*HISN2 was solved by SAD using protein crystallized in the presence of 100 µM ZnCl_2_, using the same data as for the *Mt*HISN2 unliganded structure refinement (PDB ID: 7BGM). Notably, other *Mt*HISN2 crystals were also soaked with selenourea crystal, as proposed by Luo^59^, but no selenourea molecules were found upon inspection of the final electron density maps. The phasing was performed with *Phenix.Autosol*^60^. The initial model was built using *Phenix*.*AutoBuild*^61^, and was placed inside the unit cell with the *ACHESYM* server^62^. *COOT*^63^ was used for manual model corrections between rounds of automatic model refinement in *Phenix.Refine*^64^. The nearly finished model of *Mt*HISN2 served to solve the AMP complex by molecular replacement with *PHASER*^65^. The refinement statistics are listed in Table 1.

### Kinetic measurements

Steady-state kinetic measurements were performed at 22 °C according to the method developed by Ames et al.^66^, with the Agilent 8453 spectrophotometer equipped with 8-cell automatic sample changer. Prior to experiments with *Mt*HISN2, the reaction mixture for PR-ATP production (R1) contained the kinetic buffer (4 mM Mg^2+^, 25 mM Hepes pH 7.5, 50 mM NaCl, 100 mM KCl, 1 mM TCEP), 1.3 μM *Sc*HIS1 (subunits concentration), 2.5 μM *A. thaliana* inorganic pyrophosphatase^67^, 1 mM ATP and 1 mM PRPP; however, PRPP was added immediately after blanking to start the reaction. PR-ATP formation in R1 was monitored at λ = 290 nm during 60-min incubation (Fig. 7A). The PR-ATP concentration reached 180 μM, based on the absorption coefficient, ε290 = 3600 M^−1^ cm^−1^.

The inhibition assay was performed in five cuvettes simultaneously; their content together with the experiment result is shown in Fig. 7B. Before the reaction, the cuvettes containing 900 µL of the kinetic buffer + /− AMP and/or histidine, both at 100 µM (final concentration) and wild-type MtHISN2 at 19 nM (f.c.) were incubated for 30 min. The control cuvette did not contain *Mt*HISN2. To start the reaction, 100 μl of the R1 mixture (PR-ATP) was added, the initial PR-ATP concentration was ~ 18 μM. The reaction progress was measured by monitoring ProFAR formation at λ = 300 nm^44^.

Comparative activity assay of *Mt*HISN2 mutants was performed using 790 μL of kinetic buffer to which 200 μl of the R1 mixture was added. The reactions were started by adding 10 μl of 1 mg/ml solutions of *Mt*HISN2 variants. The control cuvette did not contain *Mt*HISN2. The assay was performed in eight 1-ml cuvettes simultaneously, and the reaction progress was monitored at λ = 300 nm; the result is shown in Fig. 7C.

### Microcalorimetric study of the interaction between HISN2 and AMP

ITC measurements were carried out with MicroCal PEAQ-ITC (Malvern) at 298 K. Titrations of AMP (2 mM) against *Mt*HISN2 protein (kept at ≈ 100 µM concentration determined at 280 nm) were done in 25 mM HEPES buffer pH 7.5 (100 mM NaCl, 50 mM KCl, 1 mM TCEP, 4 mM MgCl_2_, 10 μM ZnCl_2_). AMP was injected in 19 aliquots of 2 µl. Raw ITC data were analyzed with the *Origin* 7.0 software (Origin-Lab) to obtain thermodynamic parameters like stoichiometry (*N*), dissociation constant (*K*_d_), and the changes in the enthalpy (Δ*H*) and entropy. One set of binding sites model was fitted to the data. Reference power was set to 5. A stirring speed of 750 rpm and spacing of 150 s was used. Experiments were performed in triplicate. To assign the AMP binding to a particular domain, analogical AMP titration measurements were carried on MtHISN2 mutants of the PRA-CH domain (K109A, T112V, S113A, H143E) as well as of the PRA-PH domain (R183E, T197V, Y240T).

### In-silico analyses and data presentation

The EFI-ESN web server^68^ served to calculate the sequence similarity network. The number of sequences (53 111) in the four included InterPro families: IPR008179, IPR021130, IPR002496, and IPR038019 was limited to the UniRef90 subset, which contained 21 942 sequences. The calculations were based on the alignment score of 50 for sequences between 70 and 1000 residues long. The figure was created in *Cytoscape* 3.3^69^; 6748 outliers were manually excluded from the figure.

Molecular figures were created in *UCSF Chimera*^70^, which also served to calculate the RMSD values for Cα atom pairs within 2-Å distance. Molecular docking was performed in *AutoDock Vina*^71^. The ligand and receptor files were prepared in *PyRx*^72^ and the *UCSF Chimera DockPrep* tool. The receptor file was based on *Mt*HISN2-AMP complex, with AMP removed. The search box was approx. 30 × 30 × 30 Å, centered at the AMP binding sites.

The *Nucleos* webserver^42^ was used to identify putative phosphate binding sites in the *Mt*HISN2 structure. The allowed RMSD for the structural matches between the *Mt*HISN2 structure and the reference mini-structures of nucleobases, carbohydrates, and phosphates was set to a default value of 0.6 Å. The results for nucleobase and carbohydrate predictions were omitted in the presentation.

*Caver 3.0.3 PyMol* plugin was used to calculate molecular tunnels in the structure of *Mt*HISN2 with following parameters: minimum probe radius = 0.9, shell depth = 10, shell radius = 8, clustering threshold = 3.5.

### Research involving plants

Studies complied with local and national regulations for using plants.

## Data Availability

PDB IDs: *Mt*HISN2, 7BGM; *Mt*HISN2-AMP complex, 7BGN. Raw X-ray diffraction data were deposited in the Macromolecular Xtallography Raw Data Repository (MX-RDR): unliganded *Mt*HISN2, https://doi.org/10.18150/WRT4WT; *Mt*HISN2-AMP complex, https://doi.org/10.18150/ELDWZ6.
